# Time Dependent Antinociceptive Effects of Morphine and Tramadol in the Hot Plate Test: Using Different Methods of Drug Administration in Female Rats 

**Published:** 2015

**Authors:** Morteza Gholami, Ehsan Saboory, Sogol Mehraban, Afsaneh Niakani, Nafiseh Banihabib, Mohamad-Reza Azad, Javid Fereidoni

**Affiliations:** a *Department of Biology, Faculty of Sciences, University of Urmia, Iran.*; b *Neurophysiology Research Center, Urmia University of Medical Sciences, Iran.*; c *Department of Biology, Faculty of Sciences, University of Gorgan, Iran.*; d *Department of English Language, Urmia University of Medical Sciences, Urmia, Iran. *

**Keywords:** Morphine, Tramadol, Antinociceptive, Hot plate, Rat

## Abstract

Morphine and tramadol which have analgesic effects can be administered acutely or chronically. This study tried to investigate the effect of these drugs at various times by using different methods of administration (intraperitoneal, oral, acute and chronic). Sixty adult female rats were divided into six groups. They received saline, morphine or tramadol (20 to 125 mg/Kg) daily for 15 days. A hot plate test was performed for the rats at the 1^st^, 8^th ^and 15^th ^days. After drug withdrawal, the hot plate test was repeated at the 17^th^, 19^th^, and 22^nd ^days. There was a significant correlation between the day, drug, group, and their interaction (P<0.001). At 1^st ^day (d1), both morphine, and tramadol caused an increase in the hot plate time comparing to the saline groups (P<0.001), while there was no correlation between drug administration methods of morphine and/or tramadol. At the 8^th^ day (d8), morphine and tramadol led to the most powerful analgesic effect comparing to the other experimental days (P<0.001). At the 15^th^ day (d15), their effects diminished comparing to the d8. After drug withdrawal, analgesic effect of morphine, and tramadol disappeared. It can be concluded that the analgesic effect of morphine and tramadol increases with the repeated use of them. Thereafter, it may gradually decrease and reach to a level compatible to d1. The present data also indicated that although the analgesic effect of morphine and tramadol is dose-and-time dependent, but chronic exposure to them may not lead to altered nociceptive responses later in life.

## Introduction

Pain is an important factor in the quality of human life both physically and emotionally. From the beginning of the human history, human beings have focused on the methods and drugs to relieve the pain. Opioids, such as morphine and codeine, have been and are still being applied to control the various types of pain in human beings as well as animals. Hyperalgesia, tolerance and physical dependence usually occur following repeated opioid administration (1-5). It has been reported that opioids cause analgesia in rodents such as the rats and mice in the hot plate test (6, 7). Tramadol, an artificial compound that derived from cyclohexanol HCl, is a centrally-acting, clinically-effective analgesic medication for the treatment of moderate to severe, acute and chronic pain (8-11), that structurally related to codeine, and morphine (12), but morphine may not relieve certain types of pain (13). Also, it is demonstrated that in special types of pain, tramadol efficacy is similar to that of morphine; some examples are the postoperative pain, and the ones such as nocigenic, neurogenic, or sympathogenic pains (14, 15). Tramadol is available as drops, capsules and sustained-release formulations for oral use which can be administered either orally, intramuscularly, intravenously, or by patient-controlled analgesia (15-18). Although morphine is the milestone drug in the treatment of severe pain, it can cause a wide range of side-effects such as respiratory depression, constipation, or sedation. These side-effects along with morphine-tolerance after chronic administration, can reduce analgesic efficacy, and is a significant clinical problem in some patients (19). While administration of tramadol is reported to be clinically safe, it results in effects that typically observed with opioid administration, such as nausea and vomiting (14, 20).Some studies indicated that tramadol dependence has been preclinically evaluated in different animal species (21, 22). Some other studies showed that the IP administration of tramadol produces a dose-dependent antinociception (23, 24) that remains unchanged after chronic administration (24). Tramadol comparing to morphine did not precipitate withdrawal symptoms, tolerance and dependence (15, 17, 25, 26). Some reports and controlled laboratory studies indicated that tramadol may be effective in relieving opioid withdrawal symptoms (27-29). Tramadol and morphine bind to μ-opioid receptors, but tramadol has several thousand times weaker affinity than morphine for these receptors (9). Tramadol analgesic effects are mediated by a combination of μ-opioid agonist effects and norepinephrine and serotonin reuptake inhibition (16, 23, 30, 31). In the behaviors triggered by morphine, various neurotransmitters, such as serotonin, dopamine, gamma-aminobutyric acid (GABA), and glutamate, are involved (32, 33). Although morphine and tramadol have been considered as antinociceptive element in many experiences, but it is demonstrated that analgesic potency of morphine is several times higher than tramadol (12). There is still a lack of knowledge on morphine- and tramadol-specific effects after long-term administration of these drugs (34). It is also demonstrated that they have a dose-dependent effect which is different in various times (12, 23, 24). With all studies about the relationship between these drugs, and their tolerance (15, 17, 25), there is no study to investigate their analgesic effect at different times with various doses in two different forms of administration (IP and PO). Therefore, this study was designed to investigate the antinociceptive effect of tramadol and morphine by using different methods (IP, PO, acute and chronic) of administrations at different time points following their use, and after their withdrawal.

## Experimental

*Animals*

Sixty adult female Wistar rats weighing 180–200 g were obtained from the Urmia University of Medical Sciences, Urmia, Iran. The rats were housed in Plexiglas cages in groups of four in controlled conditions with free access to food and water; standard 12-h dark/light cycle; temperature, 22 ± 2 °C; humidity, 60% and 80% (35). The bedding consisted of untreated wood shavings which were changed three times a week. Animals were handled according to 1975 Helsinki guidelines. The study was approved by the Ethical Committee of the Urmia University of Medical Science in the use of lab animals (approval number 89-01-32-322).

*Drugs*

The rats were randomly divided into six groups of ten to be administrated either intraperitoneally (IP) or orally (PO) by Saline, Morphine (Temad Co., Tehran, Iran), and/or Tramadol (Atlantis Life Sciences Co., Mumbai, India) separately. The rats in morphine, and tramadol groups received morphine or tramadol with additive doses (20, 27.5, 35, 37.5, 45, …, until 125 mg/Kg) once per day for 15 consecutive days. These additive doses for tramadol IP was previously used in different studies (36). While demonstrated tramadol hydrochloride exhibits an analgesic and antinociceptive potency that is lower than morphine (37), similar doses of morphine and tramadol were used in this study, because the current study mainly tried to compare the PO and IP methods at different time points following the drug administration and its withdrawal. Similar doses of both drugs can simplify their comparison concerning their effects, and their administration methods. The saline group received saline in a similar manner. All morphine, tramadol and saline administration occurred at the same time each day (1100 and 1200 hours). Morphine sulfate was dissolved in 0.9% saline, and it was freshly prepared for each use. Tramadol hydrochloride was liquid. 

*Pain assessment*

The nociceptive test was a hot plate test set at 54 ± 0.4 ^◦^C. The surface temperature was continuously monitored with a digital thermometer. Each rat was gently placed on the hot plate, and the latency to the stamping of the right hind paw (response latency) was recorded in seconds (35). This behavior was recorded at 30, 60, 90 and 120 min after administration of vehicle or drug at the 1^st ^(20 mg/Kg), 8^th ^(80 mg/Kg) and 15^th ^(125 mg/Kg) days of treatment. The hot plate test was repeated at the 17^th^, 19^th^, and 22^nd ^days of the experiment without any saline, morphine or tramadol administration. In this study, the animals that failed to step their paws within 60 s were removed from the plate (to avoid thermal injury), and they were assigned the value of 60 s. To avoid the different Estrous cycle in rats, and to minimize the effect of sex hormones fluctuation on the behavior of rats (38), all subject rats were in Metestrous period when the experiment was started at d1(39).

*Statistical analyses*

The parametric tests were used to analyze normally distributed data. The data were analyzed by the general linear models procedure (SPSS v.16.0) using experimental groups as categorical variable. To evaluate the basal measurements, repeated measures ANOVA were performed followed by multiple comparisons test (Bonferroni) if needed. Pain threshold behavior at different periods of time (min 30, 60, 90, and 120) and days (d1, d8, and d15) were considered as repeated measures for each animal. To compare the pain threshold before and after treatment cessation (d1, d8, d15 with d17, d19, d22), repeated measure ANOVA was performed followed by multiple comparisons test (Bonferroni). To compare the pain threshold behavior between groups at each day (d1, d8, d15, d17, 19, and d22), one-way ANOVA was performed followed by Bonferroni post hoc test when indicated. Results were expressed as means ± S.E. of the mean. Differences were considered to be statistically significant if P < 0.05.

## Results

**Figure 1:**
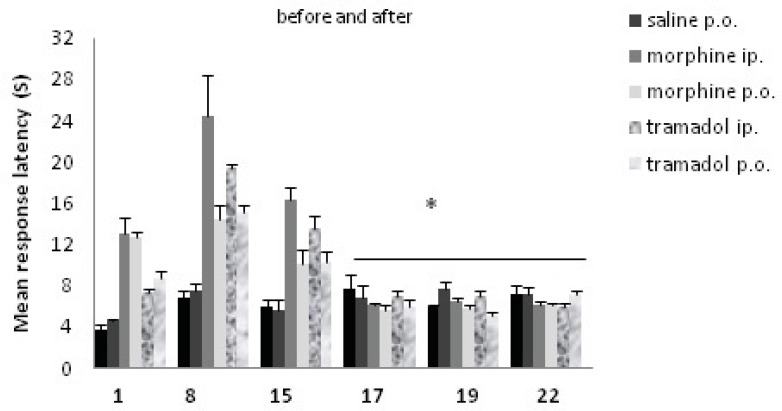
Comparisons of response latency in hot plate test during morphine and tramadol administration, and after the drugs withdrawal. The rats were treated with saline, morphine or tramadol (cumulative doses, either IP or PO) for 15 consecutive days. They were tested individually for nociceptive response in hot plate test on d1, d8, and d15 at 90 min after drug administration. Drug withdrawal took place after day 15, and nociceptive responses were tested again on d17, d19 and d22.* indicates p< 0.001(repeated measure ANOVA) with day 1, 8, and 15. There was no significant difference between days 17, 19, and 22.

**Figure 2:**
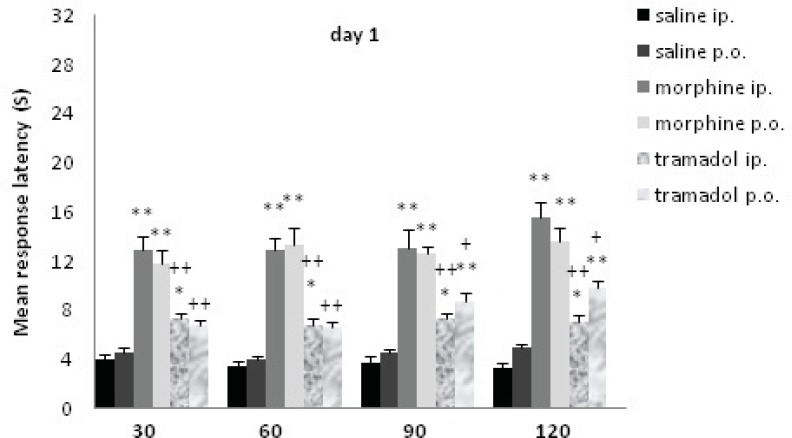
Comparisons of response latency in hot plate test every 30 min after drug administration at day1. The rats were treated with saline, morphine or tramadol (20 mg/Kg, either IP or PO), and they were tested individually for nociceptive response in hot plate test at 30, 60, 90, and 120 min after treatment. * indicates p< 0.05, ** p< 0.001 with similar saline group (*e.g*. morphine IP with saline IP); + indicates p<0.05, ++ p<0.001 with similar morphine group (*e.g.* tramadol IP with morphine IP).

Data analysis indicated that the effect of drugs was significant; there was a significant difference between before and after drug cessation. The effect of drugs significantly decreased after withdrawal [F (1, 54) =148.38, P<0.001]. Data analysis indicated that the effect of day was significant [F (2, 108) =21.76, P< 0.001]. Also, there was a significant difference on the drug × day interaction [F (2, 108) =26.62, P<0.001]. The data are represented in Figure 1. Data analysis indicated that the effect of groups was significant, the analysis revealed significant differences in nociceptive response for morphine and tramadol groups, Figure 2. [F (5, 54) =20.75, P <0.001].

At d1, the rats received 20 mg/Kg morphine or tramadol. Morphine (both IP and PO) significantly increased the time of response latency in comparison with the saline treated rats until 120 min after drug administration (p≤0.001); tramadol IP increased this variable until 120 min after injection (p<0.05), while tramadol PO just showed a non-significant increase at 30 and 60 min. It showed much more increase at 90 and 120 min that were significant comparing to saline group (p≤0.001). There was no significant difference between different methods of drug administration (tramadol IP and tramadol PO, morphine IP and morphine PO). Morphine IP showed a stronger analgesic effect comparing to tramadol IP at 30, 60, 90 and 120 min (p≤0.001) while morphine PO showed less strong effect (p≤0.05) with tramadol PO at 90 and 120 min.

**Figure 3:**
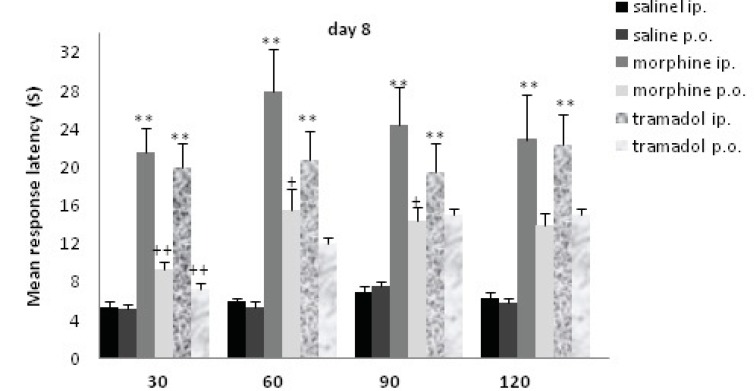
Comparisons of response latency in hot plate test every 30 min after drug administration at day 8. The rats were treated with saline, morphine or tramadol (cumulative doses, either IP or PO) for 8 consecutive days. They were tested individually for nociceptive response on hot plate test at 30, 60, 90, and 120 min after treatment. ** indicates p< 0.005 with similar saline group (*e.g*. morphine IP with saline IP); + indicates p<0.05, ++ p<0.001 with alternative method of administration (*e.g*. morphine IP with morphine PO).

The rats received additive doses of morphine or tramadol (20 to 80 mg/Kg) from d1 to d8. Morphine and tramadol IP significantly increased the time of response latency in comparison with the saline IP treated rats up to120 min after drug administration (p<0.005); while there was no significant increase for PO groups. There was a significant difference between method of drug administration at d8 (IP and PO). There was a significant difference between morphine IP and PO until 90 min (p<0.05), but concerning tramadol IP and PO, there was a significant difference at 30 min (p<0.001). There was no significant difference between morphine and tramadol on d8.

**Figure 4:**
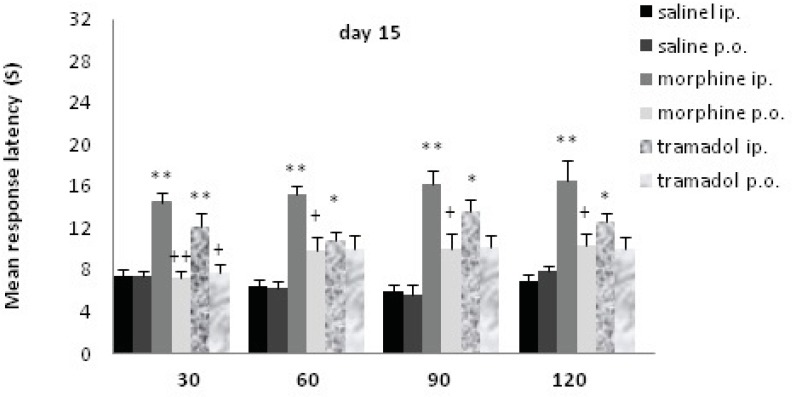
Comparisons of response latency in hot plate test every 30 min after drug administration at day15. The rats were treated with saline, morphine or tramadol (cumulative doses, either IP or PO) for 15 consecutive days. They were tested individually for nociceptive response in hot plate test at 30, 60, 90, and 120 min after treatment. * indicates p< 0.05, ** p< 0.001 with similar saline group (*e.g*. morphine IP with saline IP); + indicates p<0.05, ++ p<0.01 with alternative method of administration (*e.g*. morphine IP with morphine PO).

Following 15 days of chronic morphine or tramadol administration (20 to125 mg/Kg) a hot plate test was performed at d15. Morphine IP and tramadol IP increased the time of response latency significantly in comparison with the saline IP treated rats up to120 min after drug administration (p≤0.001with morphine; p<0.05 with tramadol); there was no significant increase for PO groups. There was a significant difference between drug administration methods at d15 (IP and PO). Morphine IP showed a significant increase comparing to morphine PO up to 120 min (p<0.01), while tramadol IP just showed a significant increase comparing to tramadol PO at 30 min(p<0.01). A significant difference was seen between morphine IP and tramadol IP just at 60 min (p<0.05).

*Drug and groups interaction*

The effect of drug*groups was significant [F (5, 54) = 30.28, P< 0.001]. Data analysis showed that the effect of day*groups was significant as well [F (10, 108) = 2.46, P= 0.011). Also, data analysis indicated that the effect of drug* day *group was significant [F (10, 108) =2.49, P=0.01).

*Nociceptive response after drug cessation*

Nociceptive response after drug cessation is represented in Table 1. There was no significant difference between the saline treated and other experimental groups. Between morphine and tramadol groups at d17, d19 and d22, there was no difference either. 

## Discussion

In the present study, the antinociceptive effect of tramadol, and morphine was investigated by using different methods of administrations at different time points. At d1 (acute dose 20 mg/Kg), while IP antinociceptive effect was greater than PO, there was no significant difference between method of drug administration (IP and PO) for morphine or tramadol groups. According to the previous findings, after intramuscular or subcutaneous injections, morphine plasma levels reach the peak in approximately 20 min, but after oral administration, its levels reach the peak in approximately 30 min(40). Following oral administration of tramadol, it is rapidly and almost completely absorbed (12). At d8 and d15, there was a significant difference between method of drug administration (IP and PO) for morphine or tramadol groups; IP administration showed an increase in the time of response latency comparing to the PO one. 

Previous findings showed that in childhood, acute low dose of morphine (5 µg/Kg) subcutaneously inducted a significant antinociceptive responses, with a peak at 30 min, while in adulthood greater doses of morphine (5 mg/Kg) IP inducted a significant antinociceptive responses, at 30 and 60 min (41). Also, it showed that in childhood repeated low dose of morphine (5 µg/Kg) subcutaneously inducted a significant antinociceptive responses, at 30, 60 and 90 min, while in adulthood repeated greater doses of morphine (5 mg/Kg) IP could not induct a significant antinociceptive responses(41). Maintained study showed that all of the age, time, dose, method and repetition may be involved on morphine antinociceptive responses. Also, it has been demonstrated that morphine and tramadol have a dose-dependent effect that varies in different time points after drug administration (41-43). In this regard, there is also evidence that, the duration of analgesic effect of a single oral dose of tramadol 100 mg is about 6 hours (44), peak analgesic effect is observed at 3.7 h (43). Our study showed that at d1 (acute), while antinociceptive effect of tramadol IP did not change at two hours, PO administration showed a time-dependent effect (in 90 and 120 min). At d8 and d15 (chronic), PO administration of morphine and tramadol showed a time-dependent increase, an increase after 30 min, and this increase for tramadol inducted a significant difference between IP and PO administration after 30 min. So, it indicated that probably tramadol PO is more time-dependent than morphine and tramadol IP. At d8, the analgesic effect of morphine and tramadol (both IP and PO) was the most powerful one comparing to the other experimental days. Additive administration of the drugs is one reason for this effect at d8 comparing to d1 (41). Also, in the rats and cats, over activation of opioid receptors have been observed to cause non-opioid receptor mediated hyperalgesia (45). There is considerable evidence that the central pharmacological effects of morphine and tramadol can be interpreted as an interaction with one or more central transmitters; the synergy between antinociception effects of monoamines and opioids is well known (46). In the rat, the threshold to nociceptive stimuli and its alteration by morphine is, in some way, depended on a dynamic balance of the concentrations of nor-adrenaline, and serotonin (5-HT) in the brain. Morphine stimulates 5-HT release via a supraspinal action (47) 5-HT depletion in the CNS decreases the analgesic effect of morphine (48), and nor-adrenaline antagonizes the antinociceptive effects of morphine. After tramadol administration, it increases the concentration of nor-adrenaline and 5-HT in brain areas (23, 31). Our data is in line with these literatures, as it showed that analgesic effect of morphine and tramadol at d8 is stronger than d1. Our study indicated that, with the increasing of doses, there may not be tolerance at d8 to these drugs, but probably at d15 there would be a tolerance to them. There are several hypotheses about how morphine tolerance develops, including opioid receptor phosphorylation which changes the receptor conformation, functional decoupling of receptors from G-proteins which leads to receptor desensitization(49), μ-opioid receptor internalization, receptor down-regulation, and up-regulation of the cAMP pathway (50). There is also evidence that long-term use of high doses of tramadol can induce physical dependence, withdrawal syndrome, and tramadol-induced tolerance (51, 52).At d17, d19, and d22, there was no antinociceptive effect for morphine or tramadol, and their effect has been disappeared. It seems that repeated morphine or tramadol administration for a moderately long period could not influence the antinociceptive effect of these drugs after their withdrawal. Therefore, there is no significant effect of either morphine or tramadol in 48 h or longer after withdrawal. By the cessation of morphine and tramadol, after 48 h, the drugs were effectively excreted from the body, and expectedly they do not show any analgesic effect. Confirming this data, morphine half-life is approximately 1.5 hours (53). Urinary excretion of morphine and conjugation of morphine in the liver leads to 83% of the drug excretion from the body after the first day of use, with 11–14% of morphine later excreted in the bile(54). While for tramadol, the central analgesic effect of tramadol 100 mg orally is only partially reversed by the opioid antagonist naloxone, while α_2_-adrenoceptor antagonism reverses tramadol effects, these point to the significant role of monoaminergic modulation and combination of μ-opioid agonist effects in tramadol antinociception after a single oral dose in human (16). In previous studies challenge dose of tramadol 24 hours after chronic doses of tramadol (15 to 120 mg/Kg, s.c.) showed that chronic tramadol was still able to reduce [^3^H]-5-HT uptake parameter by 42%. In contrast, 72 h after drug withdrawal, [^3^H]-5-HT uptake in rats chronically-treated with tramadol did not differ significantly from control rats (55). 

In conclusion, our study indicated that analgesic effect of morphine and tramadol in acute dose (20 mg/Kg) was not significantly related to the method of administration (IP or PO), but after a chronic exposure, the method of administration showed significant difference (d8 and d15). Repeated morphine and tramadol administration inducted an increase in analgesic effect, it might gradually decrease and reach to the level compatible to d1 (at d15). Therefore, analgesic effect of morphine and tramadol was time-and-dose dependent, which was more visible in tramadol PO. Meanwhile, the present data indicated that chronic exposure to opiates might not lead to an altered nociceptive response until one week after drugs withdrawal. In case of chronic use of these drugs (as a pain killer), more caution must be paid in clinical administration.
